# Reporting unit context data to stakeholders in long-term care: a practical approach

**DOI:** 10.1186/s43058-022-00369-0

**Published:** 2022-11-21

**Authors:** Lisa A. Cranley, T K T Lo, Lori E. Weeks, Matthias Hoben, Liane R. Ginsburg, Malcolm Doupe, Ruth A. Anderson, Adrian Wagg, Anne-Marie Boström, Carole A. Estabrooks, Peter G. Norton

**Affiliations:** 1 Lawrence S. Bloomberg Faculty of Nursing, University of Toronto, 155 College Street, Suite 130, Toronto, Ontario M5T 1P8 Canada; 2 University of Alberta, Edmonton, Alberta Canada; 3 School of Nursing, Dalhousie University, Halifax, Nova Scotia Canada; 4 School of Health Policy and Management, York University, Toronto, Ontario Canada; 5 Faculty of Health, York University, Toronto, Ontario Canada; 6 Rady Faculty of Health Sciences, University of Manitoba, Manitoba, Alberta Canada; 7 School of Nursing, University of North Carolina at Chapel Hill, Chapel Hill, NC USA; 8 Department of Medicine, Division of Geriatric Medicine, University of Alberta, Edmonton, Alberta Canada; 9 Department of Neurobiology, Care Sciences and Society, Division of Nursing, Karolinska Institutet, Huddinge, Sweden; 10 Theme Inflammation and Aging, Unit Aging, Karolinska University Hospital, Huddinge, Sweden; 11 R & D unit Stockholms Sjukhem, Stockholm, Sweden; 12 Faculty of Nursing, University of Alberta, Edmonton, Alberta Canada; 13 Department of Family Medicine, University of Calgary, Calgary, Alberta Canada

**Keywords:** Data reporting, Data visualisation, End-user stakeholders, Long-term care, Nursing homes, Organisational context, Quality improvement

## Abstract

**Background:**

The importance of reporting research evidence to stakeholders in ways that balance complexity and usability is well-documented. However, guidance for how to accomplish this is less clear. We describe a method of developing and visualising dimension-specific scores for organisational context (context rank method). We explore perspectives of leaders in long-term care nursing homes (NHs) on two methods for reporting organisational context data: context rank method and our traditionally presented binary method—more/less favourable context.

**Methods:**

We used a multimethod design. First, we used survey data from 4065 healthcare aides on 290 care units from 91 NHs to calculate quartiles for each of the 10 Alberta Context Tool (ACT) dimension scores, aggregated at the care unit level based on the overall sample distribution of these scores. This ordinal variable was then summed across ACT scores. Context rank scores were assessed for associations with outcomes for NH staff and for quality of care (healthcare aides’ instrumental and conceptual research use, job satisfaction, rushed care, care left undone) using regression analyses. Second, we used a qualitative descriptive approach to elicit NH leaders’ perspectives on whether the methods were understandable, meaningful, relevant, and useful. With 16 leaders, we conducted focus groups between December 2017 and June 2018: one in Nova Scotia, one in Prince Edward Island, and one in Ontario, Canada. Data were analysed using content analysis.

**Results:**

Composite scores generated using the context rank method had positive associations with healthcare aides’ instrumental research use (*p* < .0067) and conceptual research use and job satisfaction (*p* < .0001). Associations were negative between context rank summary scores and rushed care and care left undone (*p* < .0001). Overall, leaders indicated that data presented by both methods had value. They liked the binary method as a starting point but appreciated the greater level of detail in the context rank method.

**Conclusions:**

We recommend careful selection of either the binary or context rank method based on purpose and audience. If a simple, high-level overview is the goal, the binary method has value. If improvement is the goal, the context rank method will give leaders more actionable details.

**Supplementary Information:**

The online version contains supplementary material available at 10.1186/s43058-022-00369-0.

Contributions to the literature

- The need to report complex research to stakeholders in ways that balance complexity and usability is well-documented. However, methods for accomplishing this are less clear. This study presents a new method to achieve this balance using organisational context data.
- Our findings demonstrate that when feeding back complex data to stakeholders, showing more than one data visualisation method is beneficial. Stakeholders see added value in more detailed data to allow them to draw comparisons and to reveal trends.
- Involving stakeholders in evaluating data visualisation methods for reporting complex data is useful to help researchers refine data reporting methods.

## Background

Concerns about quality of care in long-term care (LTC) continue to persist [1]. Implementation of evidence into practice is a key strategy to improve care quality [2]. However, to improve care quality, evidence needs to be transformed into useable formats and mechanisms in place for uptake [1]. Tailored feedback of data is a central component of quality improvement, performance improvement, implementation, and integrated knowledge translation [3, 4]. Improving the quality of research reporting and engaging end-user stakeholders in feedback processes supports uptake of research findings [5, 6]. To optimise uptake, it is crucial to feed back research data in ways that meets stakeholders’ diverse needs [6]. Gysels and colleagues highlighted the importance of soliciting stakeholder views on feedback mechanisms and preferences for feeding back data, particularly on how research evidence is best presented to be acted upon [7].

The importance of reporting local research evidence and quality improvement data to stakeholders in ways that balance complexity and usability is well-documented [5], but methods for accomplishing this are less clear. The literature is extensive on audit and feedback reporting of data about healthcare professionals’ clinical performance [8–10] and on feedback of patient-reported outcomes [11–13]. Studies have highlighted that effectiveness (e.g. effect estimate) [8] of audit and feedback as an intervention to improve healthcare providers’ behaviours and practices, in part, depends on how feedback is provided (e.g. content, display, delivery) [8, 9, 14, 15]. For example, audit and feedback may be more effective when feedback is timely [9, 14, 16], is delivered in both verbal and written formats [8], includes measurable targets (e.g. medication prescribing behaviours, test-ordering), and has an action plan or actionable messages [8, 14, 16, 17]. Feedback should target areas for improvement and recommend actions that are under the recipient’s control [14]. Feedback should include summary descriptions for graphical displays and reports that are short, simple, and uncluttered for readability [11, 14]. For feedback to be meaningful and useful, reports must be tailored to intended audiences to meet their different decision-making needs [17]. Indeed, successful implementation of evidence into practice to improve care quality is a function of the nature and type of evidence, context, and mechanism by which change is facilitated [2].

Decades ago, Rogers’ described innovation attributes that support uptake of an innovation: relative advantage, compatibility with existing systems/practices and values of potential adopters, low complexity, trialability, and observability [18]. In accelerating uptake of research findings, the feedback mechanism by which data are fed back (e.g. facilitation) can be considered the innovation. Complex data must be presented in ways that adequately reflect the complexity of the construct being measured, while also optimising utility for stakeholders. A stakeholder’s perceptions of acceptability and appropriateness of the data presented, and the feasibility of changes required based on the data, may influence effectiveness of feedback [19]. Stakeholder involvement is crucial for successful implementation [20].

### An integrated knowledge translation approach to data feedback

Organisational context data are a complex but useful type of data to examine how to improve research reporting to stakeholders [21]. Our Translating Research in Elder Care (TREC) research programme is one example [22]. For 15 years, TREC researchers have collected data on modifiable aspects of organisational context from NH staff including nurses, unregulated healthcare aides, and allied health professionals. Data are collected from a cohort of over 90 representative NHs in Western Canada [22]. Investigators and decision-makers/end-users within TREC have a strong commitment to integrated knowledge translation. We engage our partners in all stages of the programme, working as an applied team using a context-driven approach to knowledge production [23, 24]. We feed back NHs’ and units’ own performance data on modifiable factors of organisational context. We have found that modifiable factors of organisational context such as leadership, culture, social capital (e.g. active connections among people), or organisational slack (staffing, time, space) are associated with healthcare providers’ job satisfaction [25, 26], burnout [27], best practice use [21, 28, 29], and factors that can impact quality of care and quality of life for residents (e.g. symptom burden, rushed and missed care) [30–32]. For example, we found that at the unit level, significant predictors of best practice use (e.g. protocols or guidelines) were social capital, organisational slack (staffing and time), number of informal interactions, and unit type [28]. We have also found that residents at the end of life who live in NHs with more favourable context (e.g. leadership) had significantly lower pain, shortness of breath, urinary tract infections, and lower use of antipsychotics without a diagnosis of psychosis [30]. In previous studies, we reported that healthcare aides working on care units with more favourable work environments were less likely to report rushed and missed care tasks [31, 32]. For example, Song (2020) found that healthcare aides were less likely to miss care tasks when there was increased social capital and organisational slack in staff and time and were less likely to rush care when there was increased organisational slack in staff on care units. We have found added value in reporting modifiable factors of organisational context at the care unit level, because data show between-unit variation and have greater explanatory power [21, 26, 33, 34]. Improvement priorities can be better identified and specific change initiatives implemented [35, 36]. Because NH care units are the clinical microsystems where care is provided, change strategies that are based on evidence and targeted at the unit level, rather than the NH level, are more likely to give greater success in care improvements [33, 34, 37].

### Data visualisation

Tailoring feedback data is a central component of TREC’s work. We use data visualisation tools to communicate and tailor complex data in accessible, manageable formats such as graphs, charts, and tables [38, 39]. Presenting data visually can improve understanding by allowing end-users to see patterns or trends and to compare groups, which can increase uptake for decision-making [38, 39].

We collect comprehensive survey data with the Alberta Context Tool (ACT) as one key measure. The ACT is a validated instrument developed by TREC researchers to measure modifiable aspects of organisational context. Development of the ACT survey was guided by the context domain from the Promoting Action on Research Implementation in Health Services (PARIHS) framework including leadership, culture, evaluation, and structural and electronic resources [2, 40]. In PARIHS, the terms high/low context refers to the quality of the context [2]. A higher context represents more positive contextual conditions (i.e. a more favourable context) [41]. We have used Cronbach’s alpha, factor analysis, analysis of variance, and tests of association to assess reliability and validity of the ACT in acute care [41] and LTC settings [42]. Reliability and validity of the ACT has also been assessed across healthcare settings including LTC, acute care (adult and paediatric acute hospitals), and community/home care [43]. Different versions of the ACT are available for five healthcare provider groups including nurses, healthcare aides, allied healthcare providers, specialists, and managers, and it is available in six languages [43]. Psychometric characteristics of Swedish [44] and German translations (including measurement invariance) [45] of the ACT in LTC have been reported. Previously established psychometric validation and testing of the English LTC version was based on 645 healthcare aide responses, with a Cronbach’s *α* ≥ 0.70 for 8 of the 10 ACT concepts [40]; validation is ongoing [40]. The ACT is 10-dimensional, so representing this complex construct is challenging. We have used a binary method (more favourable or less favourable organisational context factors) that we refer to as the “red/green” method [30–32]. In this binary method, individual ACT sub-scale scores are aggregated at the care unit level. K-mean clustering of each unit’s ACT scores is used to separate units and designate them as green (more favourable context) or red (less favourable context). A full description is found elsewhere [21]. The method can also use NH scores. We use scatter plots to visually display unit and facility colours in this method, although this type of representation requires an orthogonal set of variables [21]. A binary context score (0, 1) can then be used in models to assess associations between organisational context and various staff and resident outcomes [30, 32].

In a previous study, we reported that NH administrators found our presentation of results useful for assisting them to identify and implement interventions targeting areas where context was less favourable, with the goal of improving clinical outcomes [21]. However, one disadvantage of the binary method is loss of variability with respect to organisational context scores. We sometimes got questions from stakeholders such as “How do we move from red to green” and “can our unit or NH become more green or less red, or is it either/or?”

Because organisational context data are multidimensional, we continually seek ways to improve our methods while retaining maximum practicality and utility of reported data to meet stakeholder needs. Our interactions with stakeholders on their information needs and preferences on reports of survey results consistently signal the need for increasingly relevant and timely feedback [21, 46–48]. Our research team developed a new feedback method based on stakeholder feedback of wanting more information on areas to improve, for example, how to get to green (a more favourable context) on care units that were red (less favourable context). We developed what we refer to as the context rank method, which offers both more variability and different visuals. An example of development of the binary method and context rank method is detailed in Additional file 1.

### Study objectives

The objectives of this study were to:

1. Develop a more detailed method to summarise multiple aspects of organisational context (context rank method), and to provide a preliminary assessment of its association with outcome variables (criterion validity), compared to the binary method.
2. Explore perspectives of administrators and managers (hereafter leaders) from NHs on advantages, disadvantages, and utility of the binary method and context rank method for visualising and reporting complex organisational context data.

## Methods

We used a multimethod design. To address the first study objective, we retrospectively analysed ACT context data to develop an alternative method of presenting complex multidimensional data to stakeholders (context rank method). This method is more finely grained than the binary presentation yet intended to be practical for stakeholders to understand and use. To address the second study objective, we used a qualitative descriptive approach with focus groups to elicit perspectives of NH leaders on advantages, disadvantages, and utility of the binary and context rank data reporting methods [49]. Additional file 2 contains a checklist for reporting qualitative research using focus groups.

### Objective 1: A reanalysis of ACT data to obtain context rank results and preliminary criterion validity

We used the 53-item ACT data collected in 2014–2015, the most recent data available when first developing the context rank method. In addition to 10 ACT context dimensions, we included five non-ACT outcome variables from the TREC dataset in our analysis: instrumental research use, conceptual research use, job satisfaction, time rushed during care, and care left undone; as well as individual-, unit-, and facility-level variables as covariates; these measures are described in Table 1 and have been reported in our previous papers [26, 28, 31, 32, 50].

**Table 1:** List of variables: Alberta Context Tool (ACT) dimensions and non-ACT dimensions

ACT dimension*	**Definition**	**Number of items**	**Score range**
Leadership^1^	The actions of formal leaders in an organisation (unit) to influence change and excellence in practice; items generally reflect emotionally intelligent leadership.	6	1–5
Culture^1^	The way ‘we do things’ in our organisations and work units; items generally reflect a supportive work culture.	6	1–5
Evaluation^1^	The process of using data to assess group or team performance and to achieve outcomes in organisations or units.	6	1–5
Formal Interactions^2^	Formal exchanges that occur between individuals working within an organisation (unit) through scheduled activities that can promote the transfer of knowledge.	4	0–1
Informal Interactions^2^	Informal exchanges that occur between individuals working within an organisation (unit) that can promote the transfer of knowledge.	9	0–1
Social Capital^1^	The stock of active connections among people. These connections are of three types: bonding, bridging, and linking.	6	1–5
Structural Resources^3^	The structural elements of an organisation (unit) that facilitate the ability to assess and use knowledge.	7	0–1
Organisational Slack in Use of Staff^1^	Cushion of actual or potential staff resources that allows an organisation (unit) to adapt successfully to internal pressures for adjustments or to external pressures for changes.	3	1–5
Organisational Slack in Use of Time^1^	Cushion of actual or potential time resources that allows an organisation (unit) to adapt successfully to internal pressures for adjustments or to external pressures for changes.	4	1–5
Organisational Slack in Use of Space^1^	Cushion of actual or potential space resources that allows an organisation (unit) to adapt successfully to internal pressures for adjustments or to external pressures for changes.	2	1–5
**Non-ACT dimensions**^**†**^			
Instrumental Research Use^2^	The use of observable research-based practices when caring for residents (i.e. practice may be guided by guidelines, protocols, routines, care plans, or procedures that are based on research).	1	1–5
Conceptual Research Use^2^	Thinking about research-based knowledge and then using it to inform clinical decision-making.	5	1–5
Job Satisfaction^1, 5^	A global satisfaction measure that reflects affective components (i.e. feelings about one’s job).	3	1–5
Time Rushed During Care^4^	The feeling of having been rushed when carrying out necessary care tasks for residents in the last shift (e.g. bathing/toileting residents).	7	0–7
Time Leaving Care Tasks Undone^4^	Not providing residents with necessary care tasks due to a lack of time in the last shift (e.g. providing mouth care for residents, toileting residents).	10	0–10
**Non-ACT dimensions Covariates**^**†**^			
Age	Indicate age group in years; Age group (e.g. <20 years, 20–24 years, 25–29, etc)		
Sex	Indicate male or female		
English as a first language	Asked to indicate if English is their first language Yes or No		
High school completion	Asked to indicate if they completed high school Yes or No		
Burnout^6^	Emotional exhaustion	3 items	0–6
	Cynicism	3 items	0–6
	Job efficacy	3 items	0–6
Unit type	Type of unit in the nursing home (e.g. general, secure, other)		
Number of beds	Total number of resident beds in the nursing home		
Owner-operator model	Public not for profit, private for profit, or voluntary not for profit		

*Adapted with permission from Song et al. ^32^

^†^Non-ACT variables included in the TREC survey—developed by the TREC research team unless otherwise indicated

^1^Response scale: 1—strongly disagree; 2—disagree; 3—neither agree or disagree; 4—agree; 5—strongly agree; overall score: mean of items

^2^Response scale: 1—never; 2—rarely; 3—occasionally; 4—frequently; 5—almost always; scoring: recoded to 0= no interaction; 0.5= occasional interaction; 1= interaction (take count of recoded scores)

^3^Response scale: 1—never; 2—rarely; 3—occasionally; 4—frequently; 5—almost always; 6—not available; scoring: recoded to 0= no use of resource; 0.5 occasional use of resource; 1= use of resource (take count of recoded scores)

^4^Response scale: 1—yes; 2—no (response no recoded to 0; overall score is sum of items)

^5^Measured with the Michigan Organizational Assessment Questionnaire Job Satisfaction Subscale

^6^Measured with the Maslach Burnout Inventory (MBI)- General (short-form) 7-point response scale (never to daily)

Data were from 4065 unregulated healthcare aides, who comprise the largest group of direct care providers in NHs [51]. In this sample, Cronbach’s *α* was ≥ 0.70 for 8 of the 10 ACT concepts. Our data collection methods are reported elsewhere [30, 52, 53]. Healthcare aide responses from ACT data were used to derive unit context scores because they are closest to residents, providing over 90% of direct care [52]. They are uniquely positioned to evaluate the unit’s work environment (context) as it may be experienced by residents. Healthcare aides are also the only group present in sufficient numbers in NHs to obtain stable estimates when aggregating to the unit level [34].

We completed this work in two steps. First, we ranked NHs and each care unit based on scores for each of the 10 individual ACT dimensions. Development of the context rank is based on the TREC cohort data of 91 NHs and 290 care units: 33 homes from Alberta, 42 homes from British Columbia, and 16 homes from Manitoba. The average size of these homes was large (>120 beds) (mean of 129); mean number of beds ranged in size from 103 to 160 beds. NH ownership models included public (19%), private for-profit (46%), and voluntary not-for-profit (35%). We included only care units with ≥ 8 healthcare aide responses, for stable aggregation to the unit level [32, 34]. We categorised each ACT dimension scores into quartiles and labelled these as context ranks: 1 = low context, 2 = moderately low context, 3 = moderately high context, and 4 = high context. This method provides a score for one unit in relation to other units. For example, a unit leadership rank of 4 means that a care unit was a top performer for leadership (fourth quartile, top 25%) among all care units in the TREC cohort (not just among other units in an NH).

Next, we summed the 10 ACT dimension rankings to produce a single composite score for overall organisational context ranking—a context rank summary—at the NH and care unit level (range 10=low context or bottom quartile to 40=high context or top quartile) (Additional file 1). Low organisational context suggests a less favourable work environment and high organisational context suggests a more favourable work environment. We randomly selected one NH from the 91 NHs to illustrate context rank data; this home had 7 units (Table 2). The mean context rank summary for the 290 care units was 25 (SD =7, range 10–39). There were 78 (27%) units in the 25th percentile (context rank summary score 10–20), 73 (25%) units in the 50th percentile (context rank summary score 21–25), 71 (24.5%) units in the 75th percentile (context rank summary score 26–30), and 68 (23.5%) units in the 100th percentile (context rank summary score 31-39).

**Table 2:** Context rank data from a randomly selected nursing home, as shown to focus group participants

	Leadership	Culture	Evaluation	Informal interactions	Social capital	Formal interactions	Structural resources	^a^Staff	^a^Time	^a^Space	Context rank summary
Unit 1	3	2	4	4	4	2	3	1	4	4	31
Unit 2	4	2	3	3	4	1	2	2	4	3	28
Unit 3	2	2	2	4	3	3	3	2	3	3	27
Unit 4	1	1	2	3	1	1	3	4	4	4	24
Unit 5	3	3	3	3	3	2	3	1	2	3	26
Unit 6	3	3	4	3	4	2	4	1	3	2	29
Unit 7	3	2	4	4	4	4	4	4	4	4	37
Facility	3	2	3	3	4	2	3	2	4	4	30

Numbers 1–4 represent the context ranks (each ACT dimension is categorised into quartiles) where 1=low context, 2=moderately low context, 3=moderately high context, and 4=high context

The context rank summary is the sum of the 10 ACT dimension rankings to produce a single composite score for an overall organisational context ranking at the nursing home and care unit level (range 10=low context or bottom quartile to 40=high context or top quartile)

As shown in the table, care unit 7 was a top performer (high context rank of 4) in most context dimensions but had moderately high context (rank of 3) in leadership and moderately low context (rank of 2) in culture. Care unit 4 had low context (rank of 1) in 4 context dimensions: leadership, culture, social capital, and formal interactions and high context in organisational slack

^a^Organisational slack in use of staff, time, and space

The context rank method allows multiple comparisons. Organisational context performance of one care unit can be compared with other care units within the same NH and with their NH’s overall context performance. For example, as shown in Table 2, the context rank summary for care units (overall unit context score) ranged from 24 (unit 4) to 37 (unit 7). The overall NH context rank summary was 30. Similar to the binary method, the context rank method enables NH and unit level comparisons across the TREC cohort of NHs. The context rank method also allows comparisons of context ranks and composite scores. For example, organisational context performance can be compared against all other *care units* in that NH or in the TREC cohort, using unit-level context ranks for each ACT dimension and unit-level context rank summary composite scores. Organisational context performance can also be compared against all other *NHs* in our cohort, using NH level context ranks for each ACT dimension and the NH level context rank summary composite score (see Additional file 1).

We used simple linear regression analysis and included (a) the context composite score or (b) the binary method as the explanatory variable to test associations and directionality of the context rank composite scores and binary scores with outcome variables shown to have statistically significant associations in our previous research [21, 26, 31, 32]. In each regression model, covariates were selected based on statistically significant associations in our previous research (Table 1) [26, 28, 50]. Regression analysis provided *t*-test results and estimated how much variance the context composite score or binary score explained (*R*-squared statistic) [54]. SAS© software was used for data analysis.

### Objective 2: Focus groups with directors of care to compare the two methods

We sought perspectives of NH leaders on the binary and context rank methods for visualising and reporting organisational context data. We recruited NH leaders in 3 Canadian provinces that were geographically located close to researchers (LC and LW) who collected these data. As our aim was to elicit non-biased perspectives on the two reporting methods, our recruitment strategy included a purposeful sample of NHs that had not been involved in the TREC programme and leaders who had no previous exposure to our feedback methods. Eligible participants included directors of care, administrators, chief executive officers, and managers from accredited homes in the Canadian provinces of Ontario, Nova Scotia, and Prince Edward Island. We recruited leaders via email using a study invitation letter that included the study purpose, and they were followed up with a phone call by the researcher. Three focus groups with leaders were conducted in 3 NHs in person by LC and LW (who have experience conducting focus groups in LTC) between December 2017 and June 2018: one in Nova Scotia (LW), one in Prince Edward Island (LW), and one in Ontario (LC). To protect the identity of the smaller NHs in Nova Scotia and Prince Edward Island, we collectively refer to these two focus groups as the Maritimes. The sample comprised those who agreed to participate and provided written informed consent. Participants were made aware that we were TREC co-investigators. We presented an overview of the TREC study and ACT dimensions. Participants were shown a sample red/green scatter plot (binary method) based on TREC data from 36 NHs across 3 Canadian Western provinces. The scatter plot showed organisational context relative to healthcare aides reported physical and mental health status (green box high context scores, red circle low context scores) aggregated at the NH level across the three provinces (noted in the scatter plot as 1, 2, or 3). They were also shown an example of the context rank matrix using TREC data from one randomly selected NH (Table 2). Handouts of slides were provided. This gave participants background on data collected and details of the two methods for reporting context data. Focus groups lasted approximately 1 hour (including presentation of an overview of TREC).

We used a semi-structured focus group question guide to seek feedback on the two data reporting methods (Additional file 3). We elicited leaders’ perspectives on advantages, disadvantages, and utility of these two methods for receiving NH organisational context data. Guided by Roger’s innovation attributes [16], we also asked leaders to comment on each method’s understandability, meaningfulness, relevance, and usefulness to leadership teams. Focus groups were audio-recorded and transcribed, and field notes were maintained to document the context and setting. Focus group transcript data were analysed by researchers (LC and LW) using content analysis [55]. Transcripts were not returned to participants for comment or further feedback. Each focus group was first analysed independently by the researcher who conducted the focus group (LC and LW). All 3 focus group transcripts were then discussed and compared for similar descriptions and content. Data saturation was reached after the third focus group, as no new information or categories emerged [56].

## Results

### Context rank method

Means and variances of organisational context (ACT) scores of the 290 care units are shown in Table 3.

**Table 3:** Alberta Context Tool (ACT) scores of the care units (*n* = 290)

Dimension	Mean	SD	Minimum	25th percentile	Median	75th percentile	Maximum
Leadership	3.94	0.22	3.27	3.78	3.95	4.11	4.42
Culture	4.06	0.21	3.37	3.94	4.08	4.21	4.59
Evaluation	3.68	0.25	2.88	3.54	3.71	3.85	4.30
Formal interactions	1.39	0.29	0.47	1.20	1.38	1.58	2.46
Informal interactions	4.08	0.61	2.29	3.67	4.13	4.50	5.92
Social capital	4.05	0.18	3.46	3.94	4.06	4.17	4.55
Structural resources	2.70	0.78	0.81	2.18	2.72	3.23	5.04
OS—Staff	2.92	0.57	1.07	2.54	2.92	3.33	4.26
OS—Time	3.45	0.36	2.50	3.24	3.48	3.71	4.43
OS—Space	3.53	0.68	1.40	3.06	3.67	4.06	4.70

ACT scores aggregated at the unit level

Units had ≥ 8 care aide responses

*SD* standard deviation, *OS* organisational slack

Results of regression analysis are summarised in Tables 4 and 5 (see Additional file 4 for details of the full models). Results indicated that context rank summary scores had statistically significant associations with all outcome variables (*p* < 0.001); instrumental research use (*p* < .0067). The binary method had statistically significant associations with all outcome variables except for instrumental research use (*p* = 0.26). The associations were in the expected direction - positive associations with healthcare aides’ instrumental research use, conceptual research use, and job satisfaction, and negative associations with rushed care and care left undone. Coefficients of the context rank summary were consistently smaller than those of the binary method. Additionally, adjusted *R*-squared statistics of context rank models were consistently greater than those of the binary method.

**Table 4:** Regression analysis of the association between context rank summary scores and outcomes

Outcome variables	Coefficient	95% CI	*P*>|t|	Adjusted *R*^2^
Instrumental research use	0.005	0.001 to 0.009	**<.0067**	0.105
Conceptual research use	0.022	0.018 to 0.026	**<.0001**	0.568
Job satisfaction	0.016	0.012 to 0.019	**<.0001**	0.459
Rushed care	−0.063	−0.080 to −0.047	**<.0001**	0.484
Care left undone	−0.035	−0.048 to −0.023	**<.0001**	0.362

Statistically significant numbers are bolded *p*<0.05

*N* = 290 care units

Units had ≥ 8 care aide responses

CI, 95% confidence interval

**Table 5:** Regression analysis of the association between binary (red/green) scores and outcomes

Outcome variable	Coefficient	95% CI	*P*>|t|	Adjusted *R*^2^
Instrumental research use	0.029	−0.022 to 0.080	0.2609	0.085
Conceptual research use	0.182	0.119 to 0.245	**<.0001**	0.466
Job satisfaction	0.075	0.023 to 0.129	**0.005**	0.320
Rushed care	−0.528	−0.768 to −0.288	**<.0001**	0.418
Care left undone	−0.214	−0.393 to −0.035	**<.0192**	0.306

Statistically significant numbers are bolded *p*<0.05

*N* = 290 care units

Units had ≥ 8 care aide responses

CI, 95% confidence interval

### Focus groups

Sixteen leaders participated in the focus groups, representing 7 accredited not-for-profit (*n* = 4) and public (*n* = 3) NHs in Ontario, Nova Scotia, and Prince Edward Island. The number of beds per NH ranged from 40 to over 400. Participants included 3 chief executive officers, 3 directors of nursing, 5 unit managers, and 5 coordinators of services. An inclusion criterion was a minimum of 5 years of administrative or management experience in NHs. Findings from the content analysis were derived from the data.

#### Perceived advantages, disadvantages, and usefulness of the binary method

Leaders overall indicated that receiving data from both binary and context rank methods was useful. They indicated that the binary method was a good starting point, particularly to visualise data, but greater detail in the context rank method added value. Leaders identified several ways in which they believed the binary method was useful. They indicated that it was simple and easy to visualise and provided a snapshot of data. Leaders also stated that this method was useful and valuable for quickly and easily comparing data across NHs.

The visual of the colors is good - especially if you - let’s say I’m taking these back to your team on the floor, and saying, ‘This is where we’re at’…. [Ontario]

Other leaders indicated that the binary method could guide overall quality improvement plans.

…if you had areas [units] where you were red or green, it would give you decisions on where to focus some time on more quality improvement or just overall improvement plans. So, it definitely would give you some guidance. [Maritimes, FG2]

…we could do things that we do for accreditation. And I might say, ‘Okay, well I thought, you know, we are doing this, and this, and this - and maybe we do have to re-look at it’ - that’s how I would use that data… [Maritimes, FG1]

One disadvantage noted was that the binary method is not sufficiently fine-grained. Some leaders indicated that they would want to know “how green” or “how red” their NH is, and on which dimension(s) their NH scored low or high.

#### Perceived advantages, disadvantages, and usefulness of the context rank method

Leaders were uniformly positive about the context rank method. Many suggested that context rank could better inform their decision-making because it would provide comparisons within an NH and across the TREC cohort of NHs. Leaders indicated that context ranks of individual ACT dimensions would allow them to “dig deeper into the 2s or the 1s” [Ontario] and it would be useful for decisions such as education and training and planning resident care. Leaders further suggested that the context rank method could help administrators advocate for specific resources in areas where low context rank scores showed trends across time.

I think if they ran consistent - they’re not going to, I don’t think, act on anything that might just be a one time - but if it’s clear there’s a consistent kind of trend across the board then it could quite possibly [help with resources]. [Maritimes, FG1]

Leaders indicated that presenting data numerically as context ranks was advantageous to prioritise areas for improvement or change and to determine the rationale for any notable variance across units.

It [context rank] gives more detailed information - especially if it’s done by unit, it really pinpoints - it allows you to answer, so why is one unit scored so great in this area, and why is the other one scored low - something’s not working or something’s working really well here, and we’ve got to find out what they’re doing and what they’re not doing here. So, it’s a lot more detailed information that’ll help you. [Ontario]

This leader further stated:

If we look at the scores - we’d be looking at where we scored high and where we scored low. And then, looking at if some units scored better than others, why is there such a variance? What’s the rationale behind that?

Leaders suggested that we continue to report context data using the binary method only to supplement the new context rank method, because the binary method requires additional detailed explanation.

Nursing, usually they have lots of questions when we get results back from any type of survey. So yes, it would be easy to see if you were a red or a green. But then you’d want to know the breakdown of where you could improve to be a green; or a red - where you were in that - because maybe you’re a yellow…. [Maritimes, FG1]

Comparing context rank method to the binary method, one leader stated:

You could be ‘barely green’ - and you think, ‘Oh, great, we’re green. We don’t have to do anything.’ And you could be just barely red, and yet there can be one area that if you just did a little bit of work on, you wouldn’t have that issue. But the scoring [context rank] is much better, I think. [Ontario]

Leaders also found it useful to compare ranks assigned to the ACT dimensions (e.g. leadership versus staffing) within the same unit and across units in the NH. They stated that ranked context data would be easier to explain to others (e.g. Board of Directors, staff) than binary data. They noted the importance of having meetings to share study results, with a knowledgeable person communicating both results and their practical application for the NH.

When asked, participants perceived no disadvantages of the context rank method. However, one leader indicated that it would be helpful to see the wording of survey questions to assist with interpreting a score’s meaning. Leaders made other suggestions such as providing information about NH ownership status, including qualitative (narrative) data, and providing the full report to consult when needed; however, we already incorporate these strategies into our larger TREC feedback reporting activities.

## Discussion

While the binary method classifies care units into two groups (more favourable/less favourable organisational context), the context rank method assigns a numerical value as quartiles (low context, moderately low context, moderately high context, high context). Results from our regression analysis indicated that context rank summary (composite scores) and binary scores were significantly associated with outcome variables, except for a lack of association between binary method and instrumental research use. Previously, we found a positive association between organisational context factors and healthcare aides’ job satisfaction [26]. Additionally, in our previous studies using the binary method, we found positive associations with higher organisational context scores (more favourable context) and healthcare aides best practice use (instrumental and conceptual research use) [21], and negative associations with less favourable context and rushed care and care left undone [31, 32].

Both methods combine the 10 ACT subscales into a single metric to simultaneously represent multiple aspects of organisational context. However, ACT scores have different ranges and means for different dimensions. The context rank may aid stakeholder interpretation because context rank has the same range in all 10 dimensions (e.g. a context rank of 1 means the unit performed in the lowest quartile for that dimension). The context rank method shows variability in modelling and may increase explanatory power and comparisons at the care unit level. Because the context rank summary has a greater range than the binary (dichotomous) method, it represents a more refined method for reporting organisational context scores. This notion is also reflected in smaller coefficient estimates of the context rank. Most importantly, the consistently larger adjusted *R*-squared statistic in the context rank models indicates that the context rank method performs better than the binary method in modelling and that context rank better explains the variance in the outcome measures.

### Why these simplified approaches may be useful to end-users

We sought leaders’ perspectives and input to improve *relative advantage* (relevance and utility) of (1) translating our research findings into actionable knowledge for decision-making and (2) supporting practice change through quality improvement. Leaders found the binary method useful for comparisons at the care unit level and across NHs. However, they were most interested in using the context rank method to compare care units within the NH. Benchmarking can improve meaningfulness of data feedback by enabling comparisons, with the goal of continuous improvement [3, 57]. Because many factors in organisational context are modifiable, this is an alternative or additional means by which to improve quality [32].

Our focus groups discussed data visualisation methods and types of comparisons that could be made with complex context data. In TREC, we have found meetings with decision-makers to interpret study results useful, especially when direction for action may not be immediately clear [58]. Meetings may increase *observability* (potential benefits and uses) of research results, such as how our feedback reports could be made actionable for improvement by exploring change strategies. For organisational context data to be relevant and inform decision-making, leaders must understand how these data can improve work practices and outcomes [59]. Reporting performance data may not necessarily lead to its use for improvement [60, 61]. Indeed, observing change and improvements can take time, well beyond that of a research project [20]. However, aiding understanding of such data may increase uptake (and reduce the translation-to-practice gap) by facilitating interpretation and highlighting areas and strategies that might be most effective [62].

Leaders described *compatibility* of our reporting methods with current practices, such as reporting for accreditation. This familiarity was advantageous as it did not involve new learning for interpretation of results. Overall, leaders perceived the binary method as having *low complexity*—it was simple and easy to visualise. The detail of context rank method (Table 2) added value because data were perceived to be easily understandable and more readily communicated to others (e.g. how units ranked on organisational context). Although leaders in our study preferred the numbers in the context rank matrix to the binary method, they suggested that the two methods complement each other. Consistent with our findings, Hildon et al. found that tables were a well-understood and accessible means to visually display data [63]. Snyder et al. reported that end-users found red circles a good visual for identifying concerning scores immediately [64], and Brundage and colleagues found that clinicians preferred greater statistical detail for aggregated data [65]. The value in sharing both binary context data and context rank summary scores is consistent with research on performance data that highlights varying data needs of different stakeholders [66].

We are continually exploring improvements in data visualisation and reporting methods to make findings more accessible and relevant to all stakeholders. The context rank method has potential to further develop activities for engagement with feedback. Leaders in our focus groups discussed how a context rank data feedback report could inform decision-making on quality improvement activities within NHs. They perceived both binary and context rank feedback methods useful to guide quality improvement plans. However, they described the context rank method as potentially more useful for prioritising improvement areas, for resource allocation decisions, and for seeing trends or variance across units within an NH and among NHs in the TREC cohort. We will continue to evaluate these feedback methods within TREC. Further research is needed to evaluate whether research uptake (or actions taken as a result of seeing robust data) is increased when reports include end-user preferences for data visualisation and detail [67]. The ultimate question is whether these reports improve resident outcomes.

### Strengths and limitations

Strengths of the context rank method include interpretability of ranks at the ACT dimension level and variability provided by the composite score. Also, while the context rank method provides more detail to stakeholders than the binary method, both methods compare care units in the sample with each other. They are not based on absolute scores, but on sample distribution (context rank) or differences in sample scores (red/green based on K-means clustering). Therefore, units in our sample that may have low scores when compared to the total population of NH care units may still be green (or in the higher quartiles) if our unit sample has low context scores in general. Since TREC units are based on a representative stratified random sample of NHs across Western Canada, we think this risk is low. Both methods are limited in depicting absolute change over time. If units decrease or improve their context rank between two time points, that means that they have become better or worse, compared to all other units. However, if all units in our sample systematically increase or decrease their scores (e.g. due to system-level impacts such as COVID-19) while differences between units remain stable, these overall changes would not be captured by either of our two methods. Additionally, we used red and green colours for the binary method to represent traffic light colours; future considerations include using orange and blue colours to make graphs more accessible (e.g. colour blindness). Validation of the ACT is ongoing, and norm-referencing of the ACT measure is a direction for future research.

Engaging stakeholders to share their perspectives may increase relevance, quality, and usability of feedback reports. Leaders were asked for their perspectives on two data reporting methods with which they were previously unfamiliar. The methods used organisational context data that they had not previously received and required some considerable explanation. This may have made commenting more challenging for leaders. Leaders may have found both methods complementary as the scatter plot included additional data (physical and mental health) not reported in the context rank method; however, leaders did not comment on these differences in the focus group. Our qualitative sample was small, but our findings may be applicable and useful to those using data visualisation to give feedback to system stakeholders.

## Conclusions

Our organisational context data are multidimensional and complex, and a context rank method provides more explanatory power in modelling. The value of the context rank method lies in its potential to allow meaningful comparisons of variation in organisational context based on quartiles and to show trends in context ranks over time. If a simple, high-level overview is the goal, the binary method has value. If improvement is the goal, the context rank method will provide leaders with more actionable details. Providing administrators and managers of NHs with organisational context data that is more meaningful, relevant, and actionable offers greater chances for use of research data. It also offers managers additional means to identify areas for improvement and a richer toolbox for decision-making.

## Supplementary Information

**Additional file 1.** Worked examples of binary and context rank methods. An example of development of the binary method and context rank method.

**Additional file 2.** COREQ Checklist. A completed checklist for reporting qualitative research using focus groups.

**Additional file 3.** Focus group question guide. A semi-structured focus group guide used with nursing home leaders.

**Additional file 4.** Regression analysis- full models. Provides details of full models of associations between context rank summary scores and outcomes (Table 4) and associations between binary scores and outcomes (Table 5).

## Data Availability

The data supporting the conclusions of this article are housed in the secure and confidential Health Research Data Repository (HRDR) in the Faculty of Nursing at the University of Alberta (https://www.ualberta.ca/nursing/research/supports-and-services/hrdr.html), in accordance with the health privacy legislation of relevant health jurisdictions. Data specific to this manuscript can be requested through the TREC Data Management Committee (joseph.akinlawon@ualberta.ca) on the condition that researchers meet and comply with the TREC and HRDR data confidentiality policies.

## Abbreviations

NH: Nursing home
LTC: Long-term care
TREC: *Translating Research in Elder Care* research programme
ACT: Alberta Context Tool
