# Supplementary material for: Reporting unit context data to stakeholders in long-term care: a practical approach
Source: Implement Sci Commun. 2022 Nov 21;3:120. doi: 10.1186/s43058-022-00369-0 (PMC9682654; doi:10.1186/s43058-022-00369-0)
Supplement: Supplementary file 1 — Additional file 1. Worked examples of binary and context rank methods. An example of development of the binary method and context rank method. [file 43058_2022_369_MOESM1_ESM.docx]

Additional File 1.

Worked examples of binary and context rank methods.

This data was collected from the 91 nursing homes in TREC cohort in 2019-2020. Healthcare aides participated in the computer-assisted questionnaire. The data was processed at the TREC data centre, University of Alberta. The table below shows an example of a few of the cases and shows the facility-level assessments of the 10 context dimensions.

|  | Lead | Culture | Eval | IF | SC | FI | SR | OS Staff | OS Time | OS Space |
| --- | --- | --- | --- | --- | --- | --- | --- | --- | --- | --- |
| Nursing home  1 | 4.07 | 4.04 | 3.62 | 3.95 | 4.00 | 1.32 | 2.77 | 2.74 | 3.57 | 3.99 |
| Nursing home  2 | 4.14 | 3.85 | 3.71 | 3.76 | 4.33 | 1.00 | 2.37 | 2.19 | 2.84 | 4.13 |
| ….. |  |  |  |  |  |  |  |  |  |  |
|  |  |  |  |  |  |  |  |  |  |  |
| Nursing home  91 | 3.97 | 4.20 | 3.97 | 4.32 | 4.02 | 1.74 | 3.07 | 3.54 | 3.76 | 3.07 |
| Overall Mean (sd) | 3.96 (.17) | 4.05 (.16) | 3.76 (.21) | 4.17 (.48) | 4.06 (.12) | 1.47 (.26) | 2.57 (.66) | 2.82 (50) | 3.43 (.35) | 3.50 (.65) |

The ten context dimensions are labelled as Lead=Leadership, Culture, Eval=Evaluation, IF =Informal interactions, SC=Social capital, FI= Formal interactions, SR= Structural resources, OS staff= organisational slack- human resources, OS time = organisational slack- time as a resource, OS space= organisational slack- space as a resource. More details about these dimensions and their measurement can be found in Song et al. 2020.

We feed nursing home specific data back to each home together with the average performance. However, nursing homes also want more easily understood markers of their performance. To meet this need, we compute the red/green status. This is computed using k-mean clustering on the data in the table (N=91 nursing homes). Using this method, we get 2 clusters green = more favourable context and red = less favourable context. Above nursing homes 1 and 91 are green and nursing home 2 is red. Our end-users liked this approach but after a while began to ask, “How can I get to green?” or “How do I get greener?”

Hence, we developed the context rank method described in this paper. For each of the 10 dimensions of the Alberta Context Tool (ACT) we defined quartiles with 4 being the highest scores and 3 the next and so on, with 1 being the lowest scores. Each facility then had a “vector” describing its performance. Here is the result for our context rank method example.

|  | NLead | NCulture | NEval | NIF | NSC | NFI | NSR | NOS Staff | NOS Time | NOS Space | Context rank summary |
| --- | --- | --- | --- | --- | --- | --- | --- | --- | --- | --- | --- |
| Nursing home  1 | 3 | 2 | 1 | 2 | 2 | 2 | 3 | 2 | 3 | 3 | 23 |
| Nursing home  2 | 4 | 1 | 2 | 1 | 4 | 1 | 2 | 1 | 1 | 4 | 21 |
| ….. |  |  |  |  |  |  |  |  |  |  |  |
|  |  |  |  |  |  |  |  |  |  |  |  |
| Nursing home  91 | 2 | 4 | 3 | 3 | 2 | 4 | 4 | 4 | 4 | 1 | 31 |

Here NLead is the Facility’s quartile for leadership and so on. One can see where a facility might choose to try to improve. For example, nursing home 1 might choose to work at improving Evaluation, while nursing home 91 could decide to work on NOS space.
