# Supplementary material for: Reporting unit context data to stakeholders in long-term care: a practical approach
Source: Implement Sci Commun. 2022 Nov 21;3:120. doi: 10.1186/s43058-022-00369-0 (PMC9682654; doi:10.1186/s43058-022-00369-0)
Supplement: Supplementary file 2 — Additional file 2. COREQ Checklist. A completed checklist for reporting qualitative research using focus groups. [file 43058_2022_369_MOESM2_ESM.docx]

Additional File 2 COREQ 32-item Checklist

Guide Questions/ Description

Item no/ Domain

| **Domain 1: Research team and reflexivity** |  |  |
| --- | --- | --- |
| Personal Characteristics |  |  |
| 1. | Interviewer/facilitator | Which author/s conducted the interview or focus group?  **LC conducted the focus groups in Ontario and LW conducted the focus groups in the Maritimes.** |
| 2. | Credentials | What were the researcher's credentials? *E.g. PhD, MD*  **LC- RN, PhD**  **LW- Gerontologist, PhD** |
| 3. | Occupation | What was their occupation at the time of the study?  **LC- Assistant Professor**  **LW- Professor** |
| 4. | Gender | Was the researcher male or female?  **LC- female**  **LW- female** |
| 5. | Experience and training | What experience or training did the researcher have?  **LC- PhD in Nursing with experience conducting focus groups and qualitative research in long-term care.**  **LW- PhD, Gerontologist with experience conducting focus groups and qualitative research in long-term care.** |
| Relationship with participants |  |  |
| 6. | Relationship established | Was a relationship established prior to study commencement?  **No relationship**. **Long-term care homes had not been involved in the TREC program and leaders had no previous exposure to our feedback methods**. |
| 7. | Participant knowledge of the interviewer | What did the participants know about the researcher? e*.g. personal goals, reasons for doing the research*  **Reasons for doing the research were shared with participants prior to data collection (study purpose was included in the invitation letter).** |
| 8. | Interviewer characteristics | What characteristics were reported about the interviewer/facilitator? e.g. *Bias, assumptions, reasons and interests in the research topic*  **Our roles on the study (as co-investigators) were shared with participants**. |

**Domain 2: Study design**

| Theoretical framework |  |  |
| --- | --- | --- |
| 9. | Methodological orientation and Theory | What methodological orientation was stated to underpin the study? *e.g. grounded theory, discourse analysis, ethnography, phenomenology, content analysis*  **Qualitative descriptive approach. Interview questions were guided by Rogers’ (2003) Diffusion of Innovation theory**. |
| Participant selection |  |  |
| 10. | Sampling | How were participants selected? *e.g. purposive, convenience, consecutive, snowball*  **We used a purposeful sample of nursing homes and leaders who were not involved in the TREC program. We used a convenience sampling approach to recruit participants. The sample comprised those who agreed to participate and provided written informed consent.** |
| 11. | Method of approach | How were participants approached? e*.g. face-to-face, telephone, mail, email*  **Participants were recruited with a study invitation letter (which included the study purpose) by email, followed up with a phone call by a member of the research team**. |
| 12. | Sample size | How many participants were in the study?  **A total of 16 leaders participated.** |
| 13. | Non-participation | How many people refused to participate or dropped out? Reasons?  **None**. |
| Setting |  |  |
| 14. | Setting of data collection | Where was the data collected? e*.g. home, clinic, workplace*  **Data were collected in three long-term care homes**. **The focus groups were conducted in person**. |
| 15. | Presence of non-participants | Was anyone else present besides the participants and researchers?  **Non-participants were not present**. |
| 16. | Description of sample | What are the important characteristics of the sample? *e.g. demographic data, date*  **Participants included 3 chief executive officers, 3 directors of nursing, 5 unit managers, and 5 coordinators of services. An inclusion criterion was a minimum of 5 years of administrative or management experience in long-term care homes.** |
| Data collection |  |  |
| 17. | Interview guide | Were questions, prompts, guides provided by the authors? Was it pilot tested?  **A focus group interview guide was used for data collection**. |
| 18. | Repeat interviews | Were repeat interviews carried out? If yes, how many?  **Repeat interviews were not conducted in this study**. |
| 19. | Audio/visual recording | Did the research use audio or visual recording to collect the data?  **Audio-recording was used for data collection**. |
| 20. | Field notes | Were field notes made during and/or after the interview or focus group?  **Field notes were made during the focus groups**. |
| 21. | Duration | What was the duration of the interviews or focus group?  **Focus groups lasted approximately 1 hour.** |
| 22. | Data saturation | Was data saturation discussed?  **Data saturation was reached after the third focus group, as no new information or categories emerged**. |
| 23. | Transcripts returned | Were transcripts returned to participants for comment and/or correction?  **Transcripts were not returned to participants for comment.** |

**Domain 3: Analysis and findings**

| Data analysis |  |  |
| --- | --- | --- |
| 24. | Number of data coders | How many data coders coded the data?  **Two research team members (LC, LW) coded the data.** |
| 25. | Description of the coding tree | Did authors provide a description of the coding tree?  **Not applicable**. |
| 26. | Derivation of themes | Were themes identified in advance or derived from the data?  **Findings from the content analysis were derived from the data.** |
| 27. | Software | What software, if applicable, was used to manage the data?  **We did not use any software for data management based on the small number of transcripts and approach to analysis (content analysis).** |
| 28. | Participant checking | Did participants provide feedback on the findings?  **Participant checking was not conducted in this study**. |
| Reporting |  |  |
| 29. | Quotations presented | Were participant quotations presented to illustrate the themes / findings? Was each quotation identified? e*.g. participant number*  **Quotes are provided to illustrate the findings.** |
| 30. | Data and findings consistent | Was there consistency between the data presented and the findings?  **Data and findings presented are consistent**. |
| 31. | Clarity of major themes | Were major themes clearly presented in the findings?  **The perceived advantages, disadvantages, and usefulness of the two methods for reporting organisational context data are clearly presented in the findings**. |
| 32. | Clarity of minor themes | Is there a description of diverse cases or discussion of minor themes?  **We did not note any minor themes based on our analysis. of the data.** |

Tong A, Sainsbury P, Craig J. Consolidated criteria for reporting qualitative research (COREQ): A 32-item checklist for interviews and focus groups. Int J Qual Health Care. 2007;19(6):349-357.
