# Supplementary material for: Reporting unit context data to stakeholders in long-term care: a practical approach
Source: Implement Sci Commun. 2022 Nov 21;3:120. doi: 10.1186/s43058-022-00369-0 (PMC9682654; doi:10.1186/s43058-022-00369-0)
Supplement: Supplementary file 3 — Additional file 3. Focus group question guide. A semi-structured focus group guide used with nursing home leaders. [file 43058_2022_369_MOESM3_ESM.docx]

Additional File 3 Focus Group Question Guide

1. Tell us about your role is in the long-term care facility.

Prompts: job title, responsibilities, decision-making required in their role.

1. Is the red/green data useful to you in operating a long-term care facility, or a unit within a long-term care facility? Prompts: To what extent is it understandable, useful, meaningful, and/ or relevant?
2. Can you give an example of how you would use the red/green data to inform decision-making?
3. Could the red/green data be presented in a more useful way? If so, how?
4. Is the context rank data useful to you in operating a long-term care facility, or a unit within a long-term care facility? Prompts: To what extent is it understandable, useful, meaningful, and/ or relevant?
5. Can you give an example of how you would use the context rank data to inform decision-making?
6. Could the context rank data be presented in a more useful way? If so, how?
7. Which method do you prefer more: 1) red/green 2) context rank or 3) both red/green and context rank. Explain why you chose the answer you did.
8. In what format would you prefer to receive ACT data: Prompts: written report, presentation.
9. What other suggestions do you have to guide us in sharing ACT data?
10. Do you have anything else you wish to add before we finish?
