# Supplementary material for: Reporting unit context data to stakeholders in long-term care: a practical approach
Source: Implement Sci Commun. 2022 Nov 21;3:120. doi: 10.1186/s43058-022-00369-0 (PMC9682654; doi:10.1186/s43058-022-00369-0)
Supplement: Supplementary file 4 — Additional file 4. Regression analysis- full models. Provides details of full models of associations between context rank summary scores and outcomes (Table 4) and associations between binary scores and outcomes (Table 5). [file 43058_2022_369_MOESM4_ESM.docx]

Additional File 4. Regression analysis- full models

Table 4. Regression analysis of association between context rank summary scores and outcomes.

| **Outcome** | **Variable** | **Parameter Estimate** | **Standard** | **t Value** | **Pr > \|t\|** | **95% Confidence Limits** | | **Effect Size** | **R2** | **R2 Adjusted** |
| --- | --- | --- | --- | --- | --- | --- | --- | --- | --- | --- |
| **Instrumental research use** | Intercept | 4.71584 | 0.83038 | 5.68 | **<.0001** | 3.08121 | 6.35047 | 0.160 | 0.1387 | 0.1046 |
|  | Context Rank Summary | 0.00517 | 0.00189 | 2.73 | **0.0067** | 0.00145 | 0.0089 |  |  |  |
|  | Age | -0.01309 | 0.00968 | -1.35 | 0.1771 | -0.0321 | 0.00596 |  |  |  |
|  | Sex | -0.16861 | 0.38353 | -0.44 | 0.6606 | -0.9236 | 0.58639 |  |  |  |
|  | English | 0.00266 | 0.03198 | 0.08 | 0.9337 | -0.0603 | 0.06561 |  |  |  |
|  | High School | -0.09156 | 0.16346 | -0.56 | 0.5759 | -0.4133 | 0.23023 |  |  |  |
|  | MBI_Exhaustion | -0.01495 | 0.02563 | -0.58 | 0.5601 | -0.0654 | 0.03551 |  |  |  |
|  | MBI_Cynicism | -0.05483 | 0.02593 | -2.11 | **0.0353** | -0.1059 | -0.0038 |  |  |  |
|  | MBI_Efficacy | 0.08302 | 0.04533 | 1.83 | 0.0681 | -0.0062 | 0.17227 |  |  |  |
|  | Unit Type | -0.01358 | 0.00817 | -1.66 | 0.0975 | -0.0297 | 0.0025 |  |  |  |
|  | # of Beds | -0.00020857 | 0.00071 | -0.3 | 0.7681 | -0.0016 | 0.00118 |  |  |  |
|  | Operate-model | 0.01203 | 0.01643 | 0.73 | 0.4648 | -0.0203 | 0.04437 |  |  |  |
| **Conceptual research use** | Intercept | 2.7625 | 0.92492 | 2.99 | **0.0031** | 0.94177 | 4.58324 | 0.605 | 0.5848 | 0.5684 |
|  | Context Rank Summary | 0.02172 | 0.00211 | 10.3 | **<.0001** | 0.01757 | 0.02587 |  |  |  |
|  | Age | -0.01276 | 0.01078 | -1.18 | 0.2374 | -0.034 | 0.00846 |  |  |  |
|  | Sex | -0.36177 | 0.4272 | -0.85 | 0.3978 | -1.2027 | 0.47918 |  |  |  |
|  | English | 0.2377 | 0.03562 | 6.67 | **<.0001** | 0.16759 | 0.30782 |  |  |  |
|  | High School | -0.01017 | 0.18208 | -0.06 | 0.9555 | -0.3686 | 0.34825 |  |  |  |
|  | MBI_Exhaustion | 0.02239 | 0.02855 | 0.78 | 0.4336 | -0.0338 | 0.0786 |  |  |  |
|  | MBI_Cynicism | 0.02359 | 0.02888 | 0.82 | 0.4147 | -0.0333 | 0.08043 |  |  |  |
|  | MBI_Efficacy | 0.19993 | 0.0505 | 3.96 | **<.0001** | 0.10053 | 0.29933 |  |  |  |
|  | Unit Type | -0.02141 | 0.0091 | -2.35 | **0.0193** | -0.0393 | -0.0035 |  |  |  |
|  | # of Beds | 0.00103 | 0.00079 | 1.31 | 0.1902 | -0.0005 | 0.00258 |  |  |  |
|  | Operate-model | -0.01518 | 0.0183 | -0.83 | 0.4074 | -0.0512 | 0.02084 |  |  |  |
| **Job satisfaction** | Intercept | 4.81978 | 0.76798 | 6.28 | **<.0001** | 3.30799 | 6.33157 | 0.530 | 0.4798 | 0.4592 |
|  | Context Rank Summary | 0.01583 | 0.00175 | 9.04 | **<.0001** | 0.01238 | 0.01927 |  |  |  |
|  | Age | 0.02693 | 0.00895 | 3.01 | **0.0029** | 0.00931 | 0.04455 |  |  |  |
|  | Sex | -0.68214 | 0.35471 | -1.92 | 0.0555 | -1.3804 | 0.01612 |  |  |  |
|  | English | 0.03072 | 0.02957 | 1.04 | 0.2998 | -0.0275 | 0.08894 |  |  |  |
|  | High School | 0.14303 | 0.15118 | 0.95 | 0.3449 | -0.1546 | 0.44063 |  |  |  |
|  | MBI_Exhaustion | -0.09509 | 0.02371 | -4.01 | **<.0001** | -0.1418 | -0.0484 |  |  |  |
|  | MBI_Cynicism | -0.01813 | 0.02398 | -0.76 | 0.4503 | -0.0653 | 0.02907 |  |  |  |
|  | MBI_Efficacy | 0.07053 | 0.04193 | 1.68 | 0.0937 | -0.012 | 0.15306 |  |  |  |
|  | Unit Type | -0.00301 | 0.00756 | -0.4 | 0.6902 | -0.0179 | 0.01186 |  |  |  |
|  | # of Beds | -0.00001349 | 0.00065 | -0.02 | 0.9836 | -0.0013 | 0.00127 |  |  |  |
|  | Operate-model | -0.03694 | 0.01519 | -2.43 | **0.0157** | -0.0669 | -0.007 |  |  |  |
| **Rushed care** | Intercept | -0.83491 | 3.6904 | -0.23 | 0.8212 | -8.0996 | 6.42976 | 0.441 | 0.5037 | 0.4841 |
|  | Context Rank Summary | -0.06325 | 0.00841 | -7.52 | **<.0001** | -0.0798 | -0.0467 |  |  |  |
|  | Age | 0.01288 | 0.04301 | 0.3 | 0.7647 | -0.0718 | 0.09754 |  |  |  |
|  | Sex | 2.30081 | 1.70451 | 1.35 | 0.1782 | -1.0546 | 5.6562 |  |  |  |
|  | English | -0.51492 | 0.14212 | -3.62 | **0.0003** | -0.7947 | -0.2352 |  |  |  |
|  | High School | 0.14923 | 0.72648 | 0.21 | 0.8374 | -1.2809 | 1.57933 |  |  |  |
|  | MBI_Exhaustion | 0.60587 | 0.11393 | 5.32 | **<.0001** | 0.3816 | 0.83014 |  |  |  |
|  | MBI_Cynicism | -0.03903 | 0.11522 | -0.34 | 0.735 | -0.2659 | 0.18778 |  |  |  |
|  | MBI_Efficacy | -0.22777 | 0.20148 | -1.13 | 0.2592 | -0.6244 | 0.16885 |  |  |  |
|  | Unit Type | 0.03619 | 0.03631 | 1 | 0.3198 | -0.0353 | 0.10766 |  |  |  |
|  | # of Beds | 0.01036 | 0.00314 | 3.3 | **0.0011** | 0.00417 | 0.01654 |  |  |  |
|  | Operate-model | 0.24345 | 0.07301 | 3.33 | **0.001** | 0.09973 | 0.38718 |  |  |  |
| **Care left undone** | Intercept | 3.2865 | 2.80346 | 1.17 | 0.2421 | -2.2322 | 8.80521 | 0.324 | 0.3862 | 0.3619 |
|  | Context Rank Summary | -0.03521 | 0.00639 | -5.51 | **<.0001** | -0.0478 | -0.0226 |  |  |  |
|  | Age | -0.03577 | 0.03267 | -1.09 | 0.2745 | -0.1001 | 0.02854 |  |  |  |
|  | Sex | 0.13301 | 1.29486 | 0.1 | 0.9183 | -2.416 | 2.68198 |  |  |  |
|  | English | -0.16399 | 0.10796 | -1.52 | 0.1299 | -0.3765 | 0.04853 |  |  |  |
|  | High School | 0.37574 | 0.55188 | 0.68 | 0.4965 | -0.7107 | 1.46213 |  |  |  |
|  | MBI_Exhaustion | 0.28694 | 0.08655 | 3.32 | **0.001** | 0.11657 | 0.45731 |  |  |  |
|  | MBI_Cynicism | 0.07284 | 0.08753 | 0.83 | 0.406 | -0.0995 | 0.24514 |  |  |  |
|  | MBI_Efficacy | -0.51161 | 0.15305 | -3.34 | **0.0009** | -0.8129 | -0.2103 |  |  |  |
|  | Unit Type | 0.03489 | 0.02758 | 1.27 | 0.2069 | -0.0194 | 0.08919 |  |  |  |
|  | # of Beds | 0.0091 | 0.00239 | 3.81 | **0.0002** | 0.0044 | 0.0138 |  |  |  |
|  | Operate-model | 0.16763 | 0.05546 | 3.02 | **0.0027** | 0.05845 | 0.27682 |  |  |  |

**Legend**

Statistically significant numbers are bolded p<0.05

N=290 care units

Units had ≥ 8 care aide responses

Lower CL= 95% confidence interval lower limit

Upper CL= 95% confidence interval upper limit

Effect size= Cohen’s D

Table 5. Regression analysis of association between binary (red/green) scores and outcomes.

| **Outcome** | **Variable** | **Parameter Estimate** | **Standard Error** | **t Value** | **Pr > \|t\|** | **95% Confidence Limits** | | **Effect Size** | **R2** | **R2**  **Adjusted** |
| --- | --- | --- | --- | --- | --- | --- | --- | --- | --- | --- |
| **Instrumental research use** | Intercept | 4.79058 | 0.839 | 5.71 | **<.0001** | 3.13897 | 6.44219 | 0.066 | 0.1196 | 0.0847 |
|  | Binary (Red/Green) | 0.02942 | 0.02612 | 1.13 | 0.2609 | -0.022 | 0.08083 |  |  |  |
|  | Age | -0.01549 | 0.00974 | -1.59 | 0.1128 | -0.0347 | 0.00368 |  |  |  |
|  | Sex | -0.17239 | 0.3881 | -0.44 | 0.6572 | -0.9364 | 0.59159 |  |  |  |
|  | English | 0.02201 | 0.03193 | 0.69 | 0.4912 | -0.0409 | 0.08488 |  |  |  |
|  | High School | -0.13435 | 0.16553 | -0.81 | 0.4177 | -0.4602 | 0.19149 |  |  |  |
|  | MBI_Exhaustion | -0.02873 | 0.02546 | -1.13 | 0.2601 | -0.0789 | 0.02139 |  |  |  |
|  | MBI_Cynicism | -0.05202 | 0.02623 | -1.98 | **0.0483** | -0.1037 | -0.0004 |  |  |  |
|  | MBI_Efficacy | 0.10192 | 0.04543 | 2.24 | **0.0257** | 0.01248 | 0.19135 |  |  |  |
|  | Unit Type | -0.0123 | 0.00827 | -1.49 | 0.1378 | -0.0286 | 0.00397 |  |  |  |
|  | # of Beds | -0.00024711 | 0.00072 | -0.34 | 0.7326 | -0.0017 | 0.00118 |  |  |  |
|  | Operate-model | 0.01176 | 0.0167 | 0.7 | 0.4817 | -0.0211 | 0.04463 |  |  |  |
| **Conceptual research use** | Intercept | 3.04001 | 1.02837 | 2.96 | **0.0034** | 1.01564 | 5.06438 | 0.334 | 0.4861 | 0.4658 |
|  | Binary (Red/Green) | 0.18201 | 0.03201 | 5.69 | **<.0001** | 0.11899 | 0.24502 |  |  |  |
|  | Age | -0.02184 | 0.01193 | -1.83 | 0.0683 | -0.0453 | 0.00165 |  |  |  |
|  | Sex | -0.33657 | 0.47569 | -0.71 | 0.4798 | -1.273 | 0.59984 |  |  |  |
|  | English | 0.29595 | 0.03914 | 7.56 | **<.0001** | 0.2189 | 0.373 |  |  |  |
|  | High School | -0.12334 | 0.20289 | -0.61 | 0.5437 | -0.5227 | 0.27605 |  |  |  |
|  | MBI_Exhaustion | -0.02276 | 0.03121 | -0.73 | 0.4664 | -0.0842 | 0.03867 |  |  |  |
|  | MBI_Cynicism | 0.03047 | 0.03215 | 0.95 | 0.3441 | -0.0328 | 0.09376 |  |  |  |
|  | MBI_Efficacy | 0.25967 | 0.05569 | 4.66 | **<.0001** | 0.15005 | 0.36929 |  |  |  |
|  | Unit Type | -0.01809 | 0.01013 | -1.79 | 0.0752 | -0.038 | 0.00185 |  |  |  |
|  | # of Beds | 0.00061734 | 0.00089 | 0.7 | 0.4864 | -0.0011 | 0.00236 |  |  |  |
|  | Operate-model | -0.02037 | 0.02046 | -1 | 0.3203 | -0.0607 | 0.01991 |  |  |  |
| **Job satisfaction** | Intercept | 5.05715 | 0.86073 | 5.88 | **<.0001** | 3.36277 | 6.75153 | 0.166 | 0.3457 | 0.3198 |
|  | Binary (Red/Green) | 0.07578 | 0.02679 | 2.83 | **0.005** | 0.02304 | 0.12853 |  |  |  |
|  | Age | 0.01938 | 0.00999 | 1.94 | 0.0534 | -0.0003 | 0.03904 |  |  |  |
|  | Sex | -0.70368 | 0.39815 | -1.77 | 0.0783 | -1.4875 | 0.08008 |  |  |  |
|  | English | 0.09548 | 0.03276 | 2.91 | **0.0039** | 0.03098 | 0.15997 |  |  |  |
|  | High School | -0.00398 | 0.16982 | -0.02 | 0.9813 | -0.3383 | 0.3303 |  |  |  |
|  | MBI_Exhaustion | -0.14031 | 0.02612 | -5.37 | **<.0001** | -0.1917 | -0.0889 |  |  |  |
|  | MBI_Cynicism | -0.00837 | 0.02691 | -0.31 | 0.756 | -0.0613 | 0.0446 |  |  |  |
|  | MBI_Efficacy | 0.13305 | 0.04661 | 2.85 | **0.0046** | 0.04129 | 0.2248 |  |  |  |
|  | Unit Type | 0.0014 | 0.00848 | 0.16 | 0.8694 | -0.0153 | 0.01809 |  |  |  |
|  | # of Beds | -0.00006961 | 0.00074 | -0.09 | 0.9253 | -0.0015 | 0.00139 |  |  |  |
|  | Operate-model | -0.03676 | 0.01713 | -2.15 | **0.0327** | -0.0705 | -0.003 |  |  |  |
| **Rushed care** | Intercept | -1.64421 | 3.9156 | -0.42 | 0.6749 | -9.3522 | 6.06378 | 0.254 | 0.4406 | 0.4184 |
|  | Binary (Red/Green) | -0.52793 | 0.12189 | -4.33 | **<.0001** | -0.7679 | -0.288 |  |  |  |
|  | Age | 0.03935 | 0.04543 | 0.87 | 0.3872 | -0.0501 | 0.12879 |  |  |  |
|  | Sex | 2.22886 | 1.81124 | 1.23 | 0.2195 | -1.3366 | 5.79434 |  |  |  |
|  | English | -0.68532 | 0.14904 | -4.6 | **<.0001** | -0.9787 | -0.3919 |  |  |  |
|  | High School | 0.48107 | 0.77252 | 0.62 | 0.534 | -1.0397 | 2.0018 |  |  |  |
|  | MBI_Exhaustion | 0.73779 | 0.11882 | 6.21 | **<.0001** | 0.50388 | 0.97169 |  |  |  |
|  | MBI_Cynicism | -0.05923 | 0.12242 | -0.48 | 0.6289 | -0.3002 | 0.18175 |  |  |  |
|  | MBI_Efficacy | -0.40239 | 0.21204 | -1.9 | 0.0588 | -0.8198 | 0.01501 |  |  |  |
|  | Unit Type | 0.02644 | 0.03858 | 0.69 | 0.4936 | -0.0495 | 0.10239 |  |  |  |
|  | # of Beds | 0.01156 | 0.00337 | 3.43 | **0.0007** | 0.00492 | 0.0182 |  |  |  |
|  | Operate-model | 0.25843 | 0.07792 | 3.32 | **0.001** | 0.10504 | 0.41182 |  |  |  |
| **Care left undone** | Intercept | 2.78665 | 2.92161 | 0.95 | 0.341 | -2.9646 | 8.53794 | 0.138 | 0.3325 | 0.3061 |
|  | Binary (Red/Green) | -0.21424 | 0.09095 | -2.36 | **0.0192** | -0.3933 | -0.0352 |  |  |  |
|  | Age | -0.01972 | 0.0339 | -0.58 | 0.5613 | -0.0865 | 0.04701 |  |  |  |
|  | Sex | 0.14889 | 1.35145 | 0.11 | 0.9124 | -2.5115 | 2.80926 |  |  |  |
|  | English | -0.29013 | 0.1112 | -2.61 | **0.0096** | -0.509 | -0.0712 |  |  |  |
|  | High School | 0.65097 | 0.57641 | 1.13 | 0.2597 | -0.4837 | 1.78565 |  |  |  |
|  | MBI_Exhaustion | 0.37765 | 0.08866 | 4.26 | **<.0001** | 0.20312 | 0.55217 |  |  |  |
|  | MBI_Cynicism | 0.05494 | 0.09134 | 0.6 | 0.548 | -0.1249 | 0.23475 |  |  |  |
|  | MBI_Efficacy | -0.63544 | 0.15821 | -4.02 | **<.0001** | -0.9469 | -0.324 |  |  |  |
|  | Unit Type | 0.02668 | 0.02878 | 0.93 | 0.3548 | -0.03 | 0.08334 |  |  |  |
|  | # of Beds | 0.00942 | 0.00252 | 3.74 | **0.0002** | 0.00447 | 0.01438 |  |  |  |
|  | Operate-model | 0.17041 | 0.05814 | 2.93 | **0.0037** | 0.05595 | 0.28486 |  |  |  |

**Legend**

Statistically significant numbers are bolded p<0.05

N=290 care units

Units had ≥ 8 care aide responses

Lower CL= 95% confidence interval lower limit

Upper CL= 95% confidence interval upper limit

Effect size= Cohen’s D
